# Diabetic retinopathy as the leading cause of blindness and early predictor of cascading complications—risks and mitigation

**DOI:** 10.1007/s13167-023-00314-8

**Published:** 2023-02-13

**Authors:** Martina Kropp, Olga Golubnitschaja, Alena Mazurakova, Lenka Koklesova, Nafiseh Sargheini, Trong-Tin Kevin Steve Vo, Eline de Clerck, Jiri Polivka, Pavel Potuznik, Jiri Polivka, Ivana Stetkarova, Peter Kubatka, Gabriele Thumann

**Affiliations:** 1 Division of Experimental Ophthalmology, Department of Clinical Neurosciences, University of Geneva University Hospitals, 1205 Geneva, Switzerland; 2 Ophthalmology Department, University Hospitals of Geneva, 1205 Geneva, Switzerland; 3 Predictive, Preventive and Personalised (3P) Medicine, Department of Radiation Oncology, University Hospital Bonn, Rheinische Friedrich-Wilhelms-Universität Bonn, 53127 Bonn, Germany; 4 Clinic of Obstetrics and Gynecology, Jessenius Faculty of Medicine, Comenius University in Bratislava, 036 01 Martin, Slovakia; 5 Max Planck Institute for Plant Breeding Research, Carl-Von-Linne-Weg 10, 50829 Cologne, Germany; 6 Department of Histology and Embryology, and Biomedical Centre, Faculty of Medicine in Plzen, Charles University, Prague, Czech Republic; 7 Department of Neurology, University Hospital Plzen, and Faculty of Medicine in Plzen, Charles University, 100 34 Prague, Czech Republic; 8 Department of Neurology, University Hospital Kralovske Vinohrady, Third Faculty of Medicine, Charles University, Prague, Czech Republic; 9 Department of Medical Biology, Jessenius Faculty of Medicine, Comenius University in Bratislava, 036 01 Martin, Slovakia

**Keywords:** Predictive, preventive, and personalised medicine (3P/PPPM), Diabetes mellitus, Comorbidities, Diabetic complications, Retinopathy, Proliferative diabetic retinopathy, Blindness, Global burden, Health-to-disease transition, Primary and secondary prevention, Domino effect, Cerebral small vessel disease, Stress, ROS, Ischemic stroke, Mitochondrial injury, Cell death, Metabolic and signalling shifts, Inflammation, Neovascularisation, Analytical tools, Tear fluid, Molecular patterns, Biomarkers, Health policy

## Abstract

Proliferative diabetic retinopathy (PDR) the sequel of diabetic retinopathy (DR), a frequent complication of diabetes mellitus (DM), is the leading cause of blindness in the working-age population. The current screening process for the DR risk is not sufficiently effective such that often the disease is undetected until irreversible damage occurs. Diabetes-associated small vessel disease and neuroretinal changes create a vicious cycle resulting in the conversion of DR into PDR with characteristic ocular attributes including excessive mitochondrial and retinal cell damage, chronic inflammation, neovascularisation, and reduced visual field. PDR is considered an independent predictor of other severe diabetic complications such as ischemic stroke. A “domino effect” is highly characteristic for the cascading DM complications in which DR is an early indicator of impaired molecular and visual signaling. Mitochondrial health control is clinically relevant in DR management, and multi-omic tear fluid analysis can be instrumental for DR prognosis and PDR prediction. Altered metabolic pathways and bioenergetics, microvascular deficits and small vessel disease, chronic inflammation, and excessive tissue remodelling are in focus of this article as evidence-based targets for a predictive approach to develop diagnosis and treatment algorithms tailored to the individual for a cost-effective early prevention by implementing the paradigm shift from reactive medicine to predictive, preventive, and personalized medicine (PPPM) in primary and secondary DR care management.

## Preamble

**Fig. 1:**
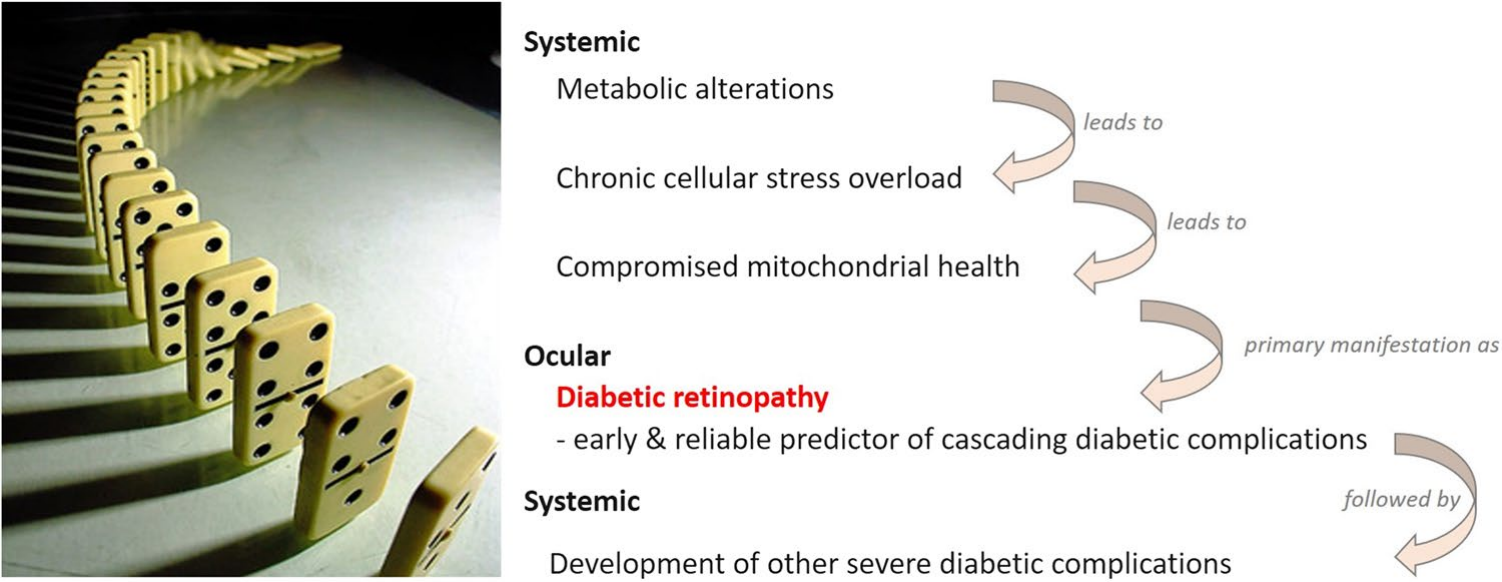
Diabetic retinopathy as an early event and a reliable predictor of severe complications linked to DM: the “domino” effect is characteristic for cascading diabetes-associated complications

Diabetic retinopathy (DR) is, together with chronic kidney disease and peripheral neuropathy, the most frequent complication of diabetes mellitus (DM) [1] and the leading cause of blindness in the working-age population [2]. Current screening processes for the risk to develop DR are not sufficiently effective allowing the disease to progress undetected until irreversible damage has occurred [3–5]. DR is clinically defined as a microvascular disease that involves damage of the retinal capillaries with secondary visual impairment [6]. Due to a particular sensitivity of retinal cells to oxidative stress, profound retinal changes are an early event and reliable predictor of complications linked to DM, which can be monitored by electroretinography as functional consequences of microvasculopathy [7]. Further, small vessel disease and neuroretinal changes create a vicious cycle fostering the development of proliferative diabetic retinopathy (PDR) with characteristic attributes including excessive mitochondrial and retinal cell death, chronic inflammation, neovascularisation, and impaired visual field leading to blindness [8]. Moreover, it has to be noted that PDR is considered an independent predictor of other severe diabetic complications such as ischemic stroke [9–11]. Contextually, a “domino effect” is characteristic for the cascading DM complications with a key role of DR as the early indicator of impaired molecular and visual signaling (Fig. 1) [9].

DR pathomechanisms are complex involving epi-/genetics and post-translational modifications, metabolic and signalling alterations, and subcellular and cellular alterations synergistically leading to retinal neurovascular dysfunction [8]. The hyperglycemia-induced systemic oxidative stress and redox imbalance are considered a causal risk of compromised mitochondrial health, and neural retina bioenergetics with vascular regression are reflected in DR pathogenesis [12]. Reactive oxygen species (ROS) overproduction, hyperactivation of inflammatory pathways, and increased mitochondrial and cellular death are characteristic for the DM-related retinal impairments [8].

The focus of this article is altered metabolomics and bioenergetics, microvascular and small vessel disease, chronic inflammation, and excessive tissue remodelling as evidence-based targets for a novel predictive approach with treatment algorithms tailored to the person and cost-effective early prevention to implement a paradigm shift from reactive medicine to preventive, predictive, and personalised medicine (PPPM) in primary and secondary DR care management. It will discuss the following:

(A) DR: global statistics and trends
(B) Association of DR with other diabetes-related complications
(C) Major systemic risks and targets of DR
(D) Targeted mitigation

## DR: global statistics and trends

### DM

Noncommunicable diseases (NCDs) pose serious health challenges with a steadily growing burden for patients and health care systems. The most prevalent form of chronic NCD is DM type 2 (T2D) [13]. T2D is considered a serious public health concern that is reaching alarming rates throughout the world [14]. Rapid economic growth and urbanization have resulted in a growing burden of DM in all countries, regardless of their level of economic development and epidemiological or demographic diversity [15]. In addition to exerting a negative impact on an individual’s functional capacity and quality of life, diabetes results in significant morbidity and premature mortality. Over the past few years, it has been noted that more than one-third of diabetes-related deaths have occurred in people under the age of 60 [16]. It is estimated that 12% of global health expenditures, or $727 billion, are devoted to diabetes and its complications, and similar to the number of patients suffering from diabetes, there is an unsustainable increase in this costs [17]. Diabetes is a disease that is strongly associated with both microvascular (including retinopathy, nephropathy, and neuropathy) and macrovascular complications (such as ischemic heart disease, peripheral vascular disease, and cerebrovascular disease) resulting in organ and tissue impairment in about one-third to one-half of diabetic patients. Several biochemical changes may interact of which one key change is increased protein glycation. This process has a broad range of impacts on surrounding tissues, including modification (e.g., thickening) of collagen and endothelium [18]. In DR, protein glycation can promote apoptosis of retinal pericytes, overproduction of endothelial growth factors, increased neovascularisation, and vascular inflammation, contributing to an elevated risk of microthrombosis formation, capillary blockage, and retinal ischemia [19]. Furthermore, abnormal stimulation of signaling cascades (such as the protein kinase C pathway), excessive production of ROS, and abnormal activation of hemodynamic regulation systems (such as the renin-angiotensin system) play major roles in microvascular complications [20].

### The global trends of DM and T2D

**Fig. 2:**
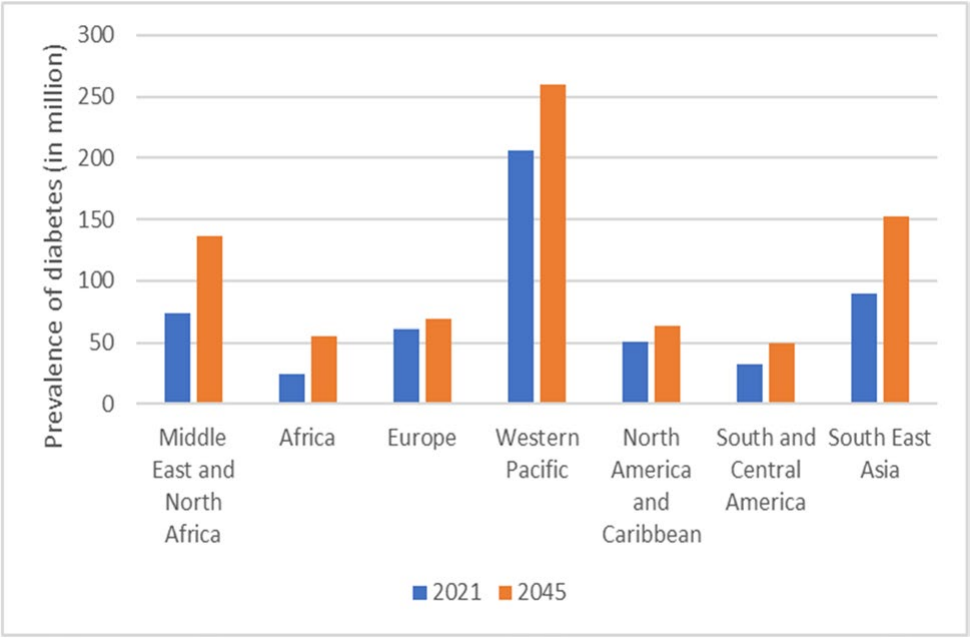
Global prevalence of DM recorded in 2021 and prognoses provided for the year 2045 [8]

Globally, DM prevalence has increased fivefold in the last four decades, from 108 million adults in 1980 to 537 million in 2021. Despite the World Health Organization’s (WHO) ambitions of reducing DM prevalence as well as premature mortality from NCDs by one-third by 2030, the outlook does not seem to be positive [21]. The number of people living with DM worldwide will increase to 643 million by 2030 and 783 million by 2045, even if age-specific prevalence remains constant [22]. In 2017, approximately 1 in 11 European adults had T2D [16], a figure projected to grow to almost 66 million by 2030 and 68 million by 2045 [23], considering that T2D accounts for 90–95% of all diabetes cases [13]. Worldwide, more than 462 million people were affected by T2D in 2017, representing 6.28% of the world’s population (4.4% of 15–49 year olds, 15% of 50–69 year olds, and 22% of 70 plus year olds) [16]. Over the past two decades, T2D-related mortality has risen dramatically; in 1990, it was the eighteenth leading cause of death, while in 2017, it ranked the ninth [21]. Moreover, it is estimated that 541 million adults suffer from impaired glucose tolerance, which puts them at high risk for developing T2D [17]. Previously confined to affluent “Western” countries, diabetes has now spread globally and has emerged as a leading cause of disability and death, even in younger age groups [24]. Over 3 in 4 adults with T2D live in low- and middle-income countries [14]. Among the International Diabetes Federation (IDF) regions, certain areas, such as the Western Pacific, have the highest number of diabetics (206 million), followed by the South-East Asia (90 million), the Middle East and North Africa (73 million), Europe (61 million), and North America and the Caribbean (51 million); South and Central America (32 million) and Africa (24 million) show the lowest number of patients [17] (Fig. 2). According to the IDF, China (140.9 million), India (74.2 million), Pakistan (33.0 million), and the USA (32.2 million) had the highest number of diabetics in 2021. In 2045, China (174.4 million) and India (124.9 million) are expected to retain the top spots of countries with the greatest number of people suffering from the disease [17].

### Prevalence of T2D in Germany

It is estimated that at least 7.2% of the German population aged 18–79 suffer from diabetes, the majority of which is T2D. The number of diabetics will increase significantly over the next two decades. A study from the German Diabetes Center in Düsseldorf and Robert Koch Institute in Berlin predicts that by 2040, Germany will have between 10.7 million and 12.3 million cases of T2D [22]. The mortality rate for people with diabetes in Germany is still twice that of people without the disease. The age-adjusted cost of direct healthcare for people with T2D is 1.7 times higher than that for those without diabetes [25].

### T2D in young populations

Young people are predisposed to T2D due to sedentary lifestyles, high-energy diets, and unknown factors, according to the IDF Diabetes Atlas [23]. Over the past few decades, obesity rates have increased, resulting in an increase in T2D cases in children, particularly in North America [17, 26]. On the basis of current incidence rates, it is projected that youth-onset T2D, defined as T2D in children and adolescents below 20 years of age, will increase fourfold by 2050 [26]. A survey conducted in 2020 found that China (520 cases/100,000 people) and the USA (212 cases/100,000 people) had the highest prevalence of youth-onset T2D, while Denmark (0.6 cases/100,000 people) and Ireland (1.2 cases/100,000 people) had the lowest prevalence [26].

### An overview of worldwide trends in DR

DR is a microvascular complication of long-term, poorly controlled DM that may cause vision-threatening damage to the retina, resulting in blindness [27]. It is one of the leading causes of preventable blindness among working-age adults [28, 29]. Among the factors that can exacerbate DR are poor glycemic control, badly managed hypertension, dyslipidemia, nephropathy, male sex, and obesity [8]. DR generally develops in distinct, consecutive phases, starting with mild non-proliferative abnormalities, characterised by elevated vascular permeability, to non-proliferative diabetic retinopathy (NPDR), defined by vascular closure, to PDR, which involves retinal and posterior vitreous surface neovascularisation [8]. DR occurs in 77.3% and 25.1% of type 1 diabetes (T1D) and T2D patients, respectively, out of which approximately 25 to 30% develop vision-threatening diabetic macular edema [27]. The growing numbers of older adults impacted by diabetes and its complications underscore the necessity of accurately monitoring DR evolution and burden. According to a 9-year follow-up study in Spain, the incidence of any DR was higher in T1D than T2D, with an annual incidence rate of 15.16, compared with 8.37, respectively [29]. The global prevalence of DR was 103 million in 2020 and is expected to reach 161 million by 2045 [30], which is primarily due to the exponentially growing population of diabetics across the globe, especially in Africa, the Middle East, North Africa, and the Western Pacific [30]. A considerable prevalence of DR was reported in Africa (35.90%) and North America and the Caribbean (33.30%) as well as the Middle East and North Africa (32.90%) [30]. The prevalence of DR for the other regions was as follows: the Western Pacific, 19.20%; Europe, 18.75%; South East Asia, 16.99%; and South and Central America, 13.37% (Table 1) [30]. The number of DR patients in the USA will reach 16.0 million by 2050, with 3.4 million suffering from vision-threatening complications [8]. The prevalence of DR among Asian patients with T2D was estimated to be 28% according to several studies [31–33]. There is a strong correlation between DR population size and DM population size, indicating a need for more resources to manage DM, as well as screen for and manage DR, particularly in the aforementioned areas; the growing number of people with DM with complications such as DR calls for special attention. Epidemiological studies demonstrated that the incidence of vision-threatening stages of DR is declining in high-income countries due to advances in therapies and improved management of diabetes [34].

**Table 1:** Prevalence and number of adults with DR in 2020 [30]

World region	Prevalence (%)	Number (in million)
Africa	35.90 (29.48–42.87)	6.99 (5.73–8.33)
North America and Caribbean	33.30 (25.29–42.40)	15.89 (12.03–20.16)
Middle East and North Africa	32.90 (26.06–40.55)	18.07 (14.28–22.28)
Western Pacific	19.20 (14.16–25.50)	31.50 (22.97–41.56)
Europe	18.75 (13.69–25.12)	11.25 (8.12–14.93)
South East Asia	16.99 (14.13–20.28)	14.95 (12.42–17.81)
South and Central America	13.37 (6.13–26.74)	4.47 (1.93–8.51)
Worldwide	22.27 (19.73–25.03)	103.12 (91.34–115.90)

### Magnitude of moderate and severe vision impairment to blindness due to DR

The WHO published a world report on vision in 2019 that summarised information from population-based assessments of visual loss and its causes worldwide [35]. In this report, DR was listed as one of the major causes of blindness and low vision. The researchers pointed out that DR is identified as the leading cause of blindness in working-age people in developed countries and is rapidly increasing in urban areas of low- and middle-income countries. The probability of developing irreversible blindness increases with increased DR severity [36], and approximately 11% of T2D patients with DR develop vision-threatening stages each year, making it a public health concern [37]. Data about prevalences of global and regional blindness and low vision estimates were updated in 2021 based on new population-based studies [30]. The prevalence of DR in diabetic patients increased to 22.27%, and it was classified as the fifth leading cause of blindness globally, with significant regional differences reaching 17% for North America and Australia, 17–15% for Europe, and 3–7% for the rest of the world [30, 38]. According to a meta-analysis of epidemiological data published in 2017 and 2021 [2, 30], 2.2 billion people in the world have vision impairment of which 3.12 million suffer from DR. The largest number of diabetics suffering from moderate to severe vision impairment due to DR was found in South Asia (1.450 million), ensued by North Africa/Middle East (336,000), Eastern Asia (279,000), Western Europe (225,000), Western Sub-Saharan Africa (193,000), Eastern Europe (166,000), Eastern Sub-Saharan Africa (128,000), and Central Latin America (109,000) [39]. The Global Burden of Disease Study 2017 [40] further detailed that blindness and vision impairment were the third cause of disability in 2017.

Thus, despite the availability of effective treatment options reviewed recently, e.g., by Lin et al. [41], DR-related vision impairment and blindness are increasing globally highlighting the importance of addressing vision loss in diabetes patients. Wykoff et al. [36] studied more than 53,000 patients with newly diagnosed DR with good vision at diagnosis for the development of irreversible blindness; 1.2% of the patients became legally blind during an average follow-up time of ∼ 1.4 years from initial DR diagnosis.

We hypothesise that one reason why the multi-step strategy to reduce DR-related blindness starting with prevention to early detection to active treatment does not show satisfying effects is due to generalised guidelines and reactive medicine after diagnosis not taking sufficiently into account the individual patient.

### Prevalence of PDR

Proliferative diabetic retinopathy (PDR), characterised by retinal neovascularisation, is the most advanced form of diabetic eye disease, both for patients with T1D and T2D. In the Wisconsin epidemiologic study of DR, 83% of cases of T1D in Wisconsin, USA, developed or progressed in level of DR in 25 years, and 42% advanced to PDR [42].

**Fig. 3:**
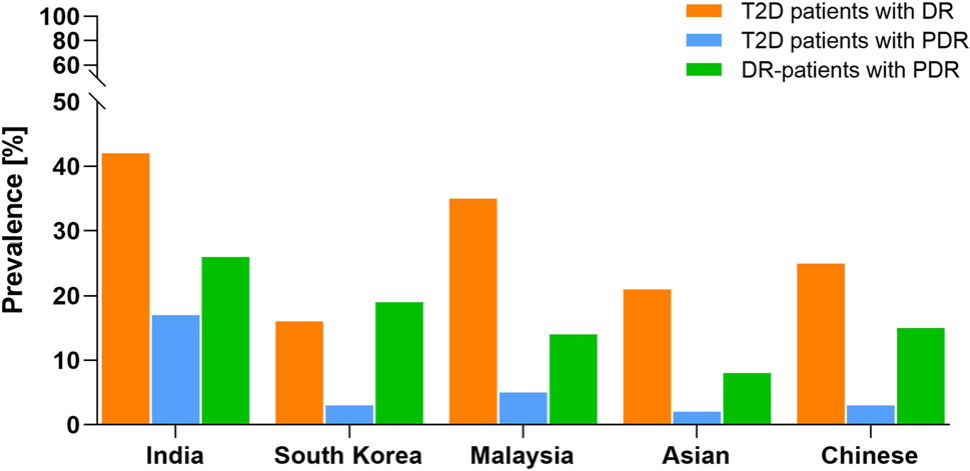
Prevalence of DR and PDR in T2D patients with and without a diagnosed DR in Asian countries [33]

A systematic review and meta-analysis performed by Yang et al. in Asian patients with T2D determined a prevalence of DR of 28% of which 6% suffered from PDR and 27% were affected by NPDR (non-proliferative DR); the prevalence of PDR and NPDR in patients already diagnosed with DR was 17% and 83%, respectively [33]. The subanalysis revealed a prevalence of PDR of 17%, 3%, 5%, 2%, and 3% in Indian, South Korean, Malaysian, Asian, and Chinese patients with T2D, respectively, compared to a prevalence of NPDR of 45%, 13%, 30%, 23%, and 22%, respectively. In T2D patients with an already diagnosed DR, the prevalence of PDR increased to 26%, 19%, 14%, 8%, and 15%, respectively [33] (Fig. 3).

In Europe, the NHS Diabetic Eye Screening Programme in England screened a total of 2,144,007 diabetics using two-field mydriatic digital photography in 2015–2016 [43].

A total of 7,593 (0.4%) urgent referrals were due to PDR and 52,597 (2.5%) to pre-proliferative diabetic retinopathy or maculopathy [43]. Overall, prevalence of DR was assessed as being 36.6% and that of PDR was 3.1% in 2016 (analysed in a subpopulation of 35,873 diabetics screened within the Gloucestershire Diabetic Eye Screening Programme) [44].

## Association of DR with other diabetes-related complications

### DR and risk of coronary artery disease

The vision impairment from DR makes managing diabetic comorbidities challenging and affects life expectancy and quality of life [43].

Generally, patients with T2D are at an increased risk of death from coronary artery disease (CAD); in T2D affected by DR, it is considered to be a contributing factor to cardiovascular events and all-cause mortality and was identified to be comparable to established risk factors, such as smoking, hypertension, and dyslipidemia in European populations [45]. DR-related vascular lesions in the eyes may reflect similar pathological disease mechanisms involved in the cerebral microcirculation [45]. As a result, having microvascular disease in the eye raises concerns about more generalised microangiopathic processes affecting the myocardium and the brain [46]. Results of a meta-analysis showed that individuals with any DR were twice as likely to experience all-cause mortality and/or cardiovascular events [47]. The meta-analysis also indicated that severe DR, either PDR or NPDR, was associated with a fourfold higher risk of composite mortality and cardiovascular disease (CVD) outcome [47]. In a study done among people with non-diabetic retinopathy and patients suffering from PDR, the risk of asymptomatic obstructive coronary artery disease was 2.16 higher in patients with PDR compared to patients affected by non-diabetic retinopathy (*P* < 0.01) [48]. Histopathological findings have indicated that DR is associated with subclinical coronary microvascular pathologic characteristics, including endothelial dysfunction, impaired coronary hypoxic-vasodilation, myocardial perfusion defects, and poorer coronary flow reserve. In diabetics, cardiac failure has been reported even in the absence of angiographically detectable CAD [45]. The pathophysiological mechanisms responsible for these findings are still controversial, but several putative factors, such as oxidative stress, inflammation, endothelial dysfunction, and advanced glycation end products (AGE), have been identified [45]. It has been shown that DR and heart failure (HF) are associated. It remains unclear how DR could contribute to the pathophysiology of HF in DM. The primary mechanism is decreased coronary microcirculation leading to chronic myocardial ischemia, which is caused by lack of compensatory angiogenesis in response to myocardial remodelling [49]. In addition, individuals with DR are more likely to have left ventricular concentric remodelling, a prerequisite for HF. According to several reports, DR reflects diastolic cardiac abnormality, a sign of HF. Hence, DR could be regarded as part of the progression of disease eventually leading to macrovascular damage and HF [50].

### DR and stroke

A stroke is the second leading cause of death among the world’s population, particularly in low- and middle-income countries [9]. In previous studies, stroke risk was typically associated with CVD; however, as further clinical studies and statistical reviews have been done, CVD no longer seems to be an appropriate explanation and predictor of stroke development [51]. As a result, studies on stroke and diabetes have received an increased interest in recent years. There is evidence that diabetes is an important risk factor for stroke; people with diabetes have a 2 to 4 times higher stroke incidence than those without the disease. Diabetes-related microvascular complications are widely acknowledged as a risk factor for the macrovascular sequelae of stroke [9]. Moreover, it has to be noted that according to the meta-analysis of 19 studies involving 45,495 patients, people with diabetes who have DR have an elevated stroke risk—a relationship that was significant for patients with T2D but inconclusive for patients with T1D [10]. Studies reviewed by Hu et al. have demonstrated that stroke risk increases as DR stage and lesion severity increase [52].

### DR and neuropathy

As the prevalence of pre-diabetes and diabetes increases worldwide, so have the complications associated with these disorders. The most common complication is neuropathy, in particular distal symmetric polyneuropathy (referred to as diabetic peripheral neuropathy, DPN). A DPN is characterised by loss of sensory function in the lower extremities, along with pain and substantial morbidity. Approximately half of individuals with diabetes develop DPN, which is associated with DM duration [53, 54]. Additionally, a significant association has been observed between DR severity and DN severity. Individuals with DPN were 4.88 times more likely to have DR than those without (41.5% vs. 8.5%); it has been also shown that nearly all PDR patients have DPN [55]. The study performed by Abougalambou and Abougalambou [56] demonstrated that DR is one of the most important risk factors for DPN; DR was observed to be 2.85 times more common in patients with DPN. Likewise, a study among the Korean population found DR to be 1.78 times more prevalent among DPN than in those without [57].

### DR and diabetic nephropathy

Diabetic nephropathy (DN) is characterised by chronic proteinuria, hypertension, and low glomerular filtration rate [58]. It is estimated that 25–45% of patients with T1D will experience nephropathy with a peak time for its development at 10–15 years after disease onset [58, 59]. Patients with T2D are less likely to develop nephropathy with a prevalence of less than 20% [59]. According to the study, 35% of T1D patients suffering from symptomatic DR also show signs of DN [58]. The mechanisms by which chronic hyperglycemia leads to microvascular disorders such as DR and DN are almost identical, so their onset and progression are tightly related [60]. In conclusion, we can predict the presence/absence and severity of DN in diabetics based on DR severity. Furthermore, PDR is significantly more common among patients with macroalbuminuria than those with microproteinuria. According to Singh et al., increased urinary albumin excretion is associated with PDR [61].

### DR and diabetic foot syndrome

Diabetic foot syndrome (DFS) is one of the major complications of long-term uncontrolled diabetes, which results from a combination of DPN and microvascular complications in the lower-limb extremities. Depending on the severity, it can range from a minor ulcer to necrosis of tissues, which may necessitate an amputation [62]. DM affects more than 25 million Americans of which 15–25% will suffer from diabetic foot ulcers (DFU) [63, 64]. There is evidence that DR is a risk factor for progressing of DFS [65] and that the severity of the DR is greater in patients with higher grades of DFS [65, 66]. Hwang et al. demonstrated the association of PDR and DFS [64]; they observed that diabetes patients without DFU had a prevalence of 4.5% of DR, while those with DFU had a 90% prevalence, with more than half exhibiting PDR. They hypothesised that it could be due to an increase in oxidative stress and endothelial damage occurring in vascular disease in the later stages of diabetes [64].

## Major systemic risks and targets of DR

### Complexity of the DR pathogenesis

Diabetic microangiopathy is the pre-stage of DR characterised by abnormal growth and leakage of small blood vessels, resulting in local edema and functional multi-organ impairments [67]. The resulting DR is characterised by pronounced neurodegenerative features. The pathogenesis is still not completely understood, but inflammatory reactions provoked by advanced glycation end products, toll-like receptors, and hyperosmolar and oxidative stress are taking part of the complex interplay of multiple mechanisms in the development of micoangiopathy and DR [67]. Corresponding indicative biomarkers able to detect cellular stress and inflammation, e.g., malondialdehyde (MDA), advanced oxidation protein products (AOPP), nitrotyrosine, and pro-apoptosis molecules (caspase-3, Fas, and Bax), are reflecting alterations in respective affected pathways and are released locally as well as into the tear fluid and circulation. The validation of these indicators and the identification of DR-predictive biomarker patterns would enable the identification of patients at risk for DR and make both body fluids particularly attractive for a predictive diagnostic approach [7]. To this end, damage and apoptosis of retinal neurons and ganglion cells would be detectable before the onset of clinical signs of microangiopathy and could be utilised for the targeted protection (see the “Major systemic risks and targets of DR” section) at the level of the health-to-disease transition [9]. Further, a dysregulated vascular regeneration plays a role in the context of oxidative and hyperosmolar stress as well as hyperactivation of inflammatory pathways [67]. Finally, objective measurements of compromised mitochondrial health quality are considered another very promising approach towards the health-to-disease transition in DM and related complication [9, 68, 69].

### Altered metabolic pathways as a risk factor and potential therapeutic targets

#### Obesity as the risk of T2D and NPDR

In 2016, prevalence rates for pre-obesity and obesity exceeded 60%; in the USA, costs related to obesity exceed 9.3% of the GDP, and in Italy, these rates exceeded 40% [70]. It was estimated that total annual health care expenditure of the Italian national health service due to “excess weight” is about €4.5 million; however, this cost seems to be an underestimation given the fact that obesity is preventable and reversible and often does not itself induce costs. More realistic is the consideration of costs of comorbidities whose risk is increased due to obesity, and indeed, the total annual cost of, e.g., diabetes alone was estimated at €20.3 billion [70]. Excessive adipose tissue and, in particular, visceral fat cause metabolic stress, organ dysfunction, and increase the risk of T2D. Obesity shows a known clear metabolic phenotype, and if recognised as a disease, individualised approaches could consider behavioral and lifestyle changes, among others, to stabilise the health status effectively protecting the affected individual against the health-to-disease transition at the level of DM pre-stage. An association between obesity and DR has still to be established [12]. To this end, if stratified by DR severity, meta-analysis of prospective studies demonstrated obesity as a risk factor of NPDR but not of PDR demonstrating a significant difference in pathomechanisms [71].

#### Hyperhomocysteinemia as a facilitator of vicious circle of mitochondrial impairment in DR

Increased homocysteine levels were demonstrated in the serum, vitreous, and retina of diabetic patients and experimental animal models of diabetes [72]. Hyperhomocysteinemia is an additional systemic metabolic risk factor associated with increased oxidative stress and, therefore, synergistic with hyperglycemia in the pathogenesis of DR [73]. Systemic effects of elevated homocysteine carry a multi-faceted character. There is a significant contribution to endothelial dysfunction and vasculopathy [74] as well as altered modification of genes, since the DNA methylation machinery is activated due to homocysteine biosynthesis involved in the S-adenosyl methionine—the co-substrate of DNA methylation [75]. Consequent systemic effects are reflected in the epigenetic modifications of chromosomal and mitochondrial DNA (mtDNA) affecting methylation of the damaged mtDNA and impaired biogenesis [68]. Thus, hyperhomocysteinemia can be seen as a facilitator of vicious cycle of mitochondrial impairment in T2D and DR. Contextually, the homocysteine metabolism would be an attractive target for the DR prevention: regulation of systemic homocysteine levels linked to transsulfuration and remethylation processes should ameliorate retinal mitochondrial and vascular damage [74, 76].

#### Endothelin-1 is associated with PDR

Endothelin-1 (ET-1) is a potent vasoconstrictor involved in the regulation of processes relevant to physical and mental well-being. Altered ET-1 homeostasis is an independent predictor of suboptimal health conditions and health-to-disease transition [77]. In the context of DR, ET-1 has been demonstrated to promote the development of PDR [78]. In fact, the mean aqueous humor ET-1 levels are significantly increased in the eyes of patients diagnosed with advanced DR compared to patients with early DR and healthy individuals [79] indicating a direct involvement of ET-1 in the local retinal pathophysiology and suggesting the use of ET-1 levels in the tear fluid as a predictive diagnostic tool [80].

### Cerebral small vessel disease is associated with the severity of DR

Cerebral small vessel disease (CSVD) is considered a common feature of DM; however, it is frequently neurologically asymptomatic in DM individuals [11]. Due to early retinal changes in DM-associated complications, specifically retinal vasculature abnormalities were hypothesised to mirror early stages of CSVD [81]. It has been shown that there is a significantly higher prevalence of PDR in T1D patients with CSVD compared to those without CSVD [11]. Further, presence of CSVD and, in particular, cerebral microbleeds was characteristic for DM patients with PDR and is therefore associated with the severity of DR [11]. Consequently, retinal microvascular abnormalities were proposed as a useful predictor of CSVD [81].

Finally, the mechanisms of small vessel disease in DR were brought into context of the lacunar stroke pathophysiology and subclinical cerebrovascular diseases [9]. The key elements are mitochondrial injury, pro-inflammatory process, and extensive tissue remodelling. Corresponding targets might be of great clinical utility for predictive diagnostic and treatment purposes.

### PDR as an independent predictor of ischemic stroke—the case presentation

**Fig. 4:**
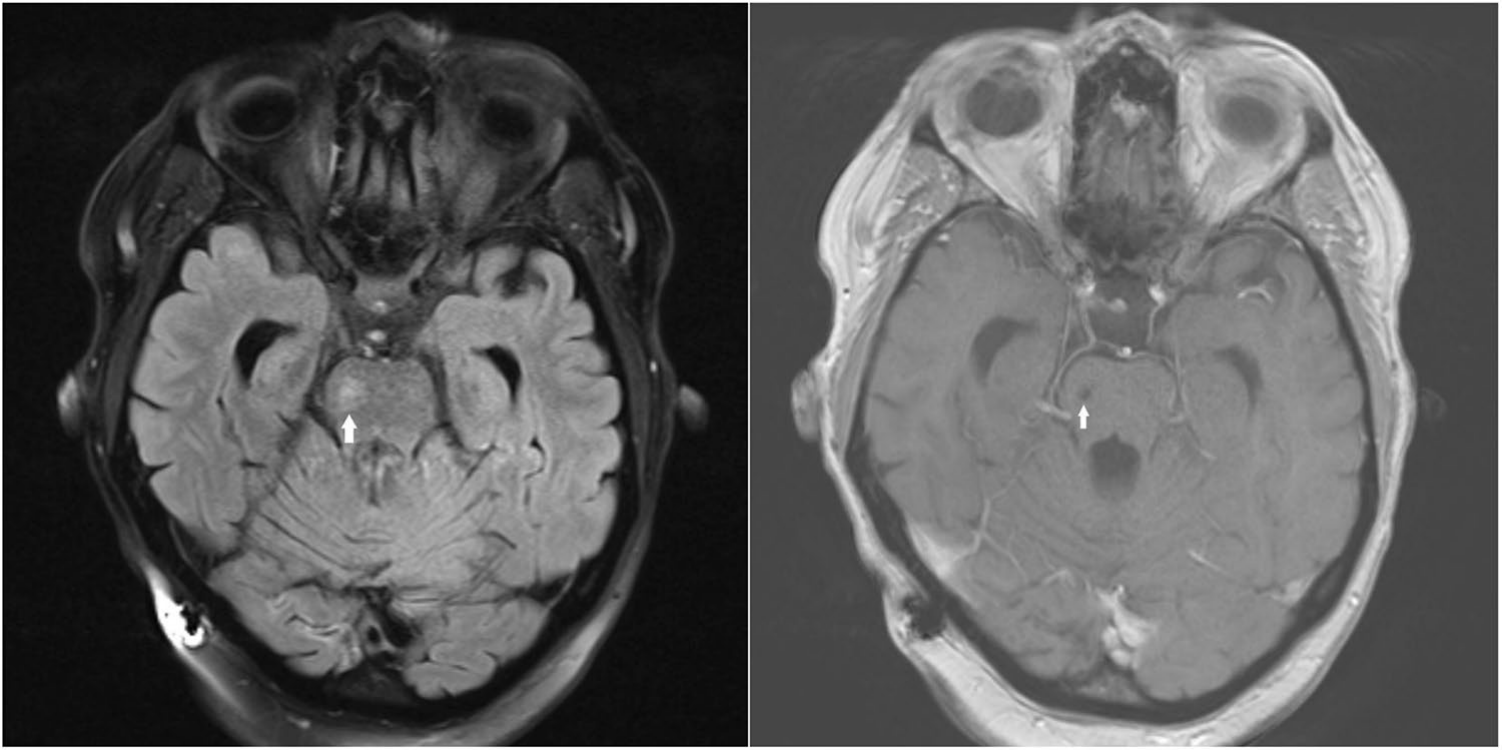
MRI revealed lacunar stroke in the right side of the pons and diffuse T2 hyperintensity and T1 hypointensity widespread lesions in the white matter of the brain (white arrows)

A 47-year-old woman was referred to hospital due to sudden onset of severe left-sided hemiparesis with saving of cranial nerve affection. Her medical history indicated that the patient was diagnosed with T1D at 15 years of age and has been treated with an insulin pump equipped with a glucose sensor. Since the onset of diabetes, she has been diagnosed with polyneuropathy; nephropathy, which has progressed to stage 3; and NPDR, which has progressed to bilateral PDR at the age of 45 years. The patient suffered with arterial hypertension, dyslipidemia, and normal pressure hydrocephalus, which has been treated with ventriculo-peritoneal drainage. At admission, multimodal CT scan, CT perfusion, and CT angiography showed no visible lesion corresponding to acute ischaemic or haemorrhagic stroke. The patient was treated with intravenous thrombolysis (Actilyse) resulting in partial recovery. The transthoracic and transesophageal echocardiography and ECG Holter were normal; however, MRI revealed lacunar stroke in the right side of the pons and diffuse T2 hyperintensity and T1 hypointensity with widespread lesions in the white matter of the brain (Fig. 4). Ethics approval FN/1252/01 dated on July 2nd 2020 has been provided by the Ethical Commission of University Hospital Plzen and Faculty of Medicine in Plzen, Charles University, Prague, Czech Republic.

## Targeted mitigation

### Current approaches to prevent DR and avoid vision loss due to DR

There are multiple strategies to reduce DR-related blindness, ranging from prevention to early detection with active treatment, if necessary. Generally, detection and treatment of eye diseases at an early stage are essential to avoid complications and risks of vision loss [33] and thus, in order to prevent the development of DR, diabetes and hypertension management is generally prioritised due to their consistent role in aggravated DR development and progression [82]. In addition, early diagnosis and management of DR are generally acknowledged as a key in reducing diabetes-related visual impairment [82]. For instance, following the implementation of a national screening program designed to identify vision-threatening DR, blindness from DR was significantly reduced in Iceland, England, and Wales [43, 44].

### Stabilised metabolism

Sustained hyperglycemia in diabetic patients leads to an accumulation of sorbitol in retinal cells by the activity of aldose reductase. Sorbitol induces osmotic stress in the retina; thereby further damaging retinal blood vessels. Continuous systemic hyperglycemia promotes non-enzymatic spontaneous glycation of proteins referred to as advanced glycation end products (AGEs) [83]. The breakdown of the blood-retinal barrier (BRB) under hyperglycemia conditions leads to leaky blood vessels and macular edema [84]. Diabetic dyslipidemia initiates retinal vascular degeneration through specific mechanisms [85]: low-grade chronic inflammation in retinal cells that result in endothelial cell injury, inadequate repair of injured retinal capillaries by bone marrow-derived circulating angiogenic cells, and activation of myeloid cells that support a pro-inflammatory environment in the retina, which are also involved in the development of DR. Hou et al. [86] evaluated dysregulations in amino acid levels as potential biomarkers. Hyperglycemia, dyslipidaemia, nitrogen metabolism, and the urea cycle as well as several metabolites including l-glutamine, l-glutamic acid, pyruvic acid, l-lactic acid, acetic acid, l-alanine, d-glucose, l-threonine, l-lysine, citrulline, and succinic acid have been associated with the pathogenesis of DR [87].

There are several clinical approaches to stabilise metabolic imbalances that can be implemented for the treatment of DR. Even though precise glycemic control is beneficial in both T1D and T2D patients [88, 89], it appears that for the treatment of DR, it is not sufficient and that additional therapies are needed [90].

Despite the lack of an unambiguous connection between DR and traditional lipid markers, lipid-lowering drugs may be effective adjunctive agents in the management of DR [91]. Data from clinical trials point to the beneficial effects of fenofibrates, statins, and candesartan on the progression of DR by controlling of dyslipidemia [91]. In this regard, fenofibrate demonstrated a significant decrease in the DR progression in individuals with pre-existing mild DR; however, it did not affect the patient’s vision and did not prevent DR development in T2D patients without DR [92]. Further large-scale and well-designed clinical trials are necessary to clarify precise mechanisms by which lipid-lowering drugs suppress the processes of DR progression.

Finally, current research confirms the role of elevated excitotoxic metabolites, including glutamate, branched-chain amino acids, and homocysteine in the pathology of DR [72, 93]. The initiation of DR is associated with a vitamin B_12_ and folic acid deficiency, which shows an association to hyperhomocysteinemia and following neurodegeneration [93]. Alterations in glutamate and other neurotransmitter levels have been reported to initiate metabolic pathways that trigger a decrease of neurotrophic factors and an increase in oxidative stress, which lead to retinal neurodegeneration in diabetic patients, whereas treatment by glutamate inhibitors appears to protect neurons [94]. Data from ongoing clinical trials will help to define the role of glutamate in ocular pathologies including DR. Further research is needed to better understand specific signalling pathways of neurodegeneration and consequently set up effective clinical strategies for neuroprotection in diabetic patients.

### Lifestyle patterns

As aforementioned, diabetes is also known as a lifestyle disorder that has become a public health burden worldwide. The progressive disorder is associated with several complications, including microvascular (DR, nephropathy, and neuropathy) and macrovascular diseases (cardiovascular, cerebrovascular, and peripheral vascular disease) [95].

A sedentary lifestyle, lack of physical activity, and bad dietary habits can lead to the development and progression of diabetes and related complications, especially in obese individuals [96, 97]. A lack of motivation represents the limitation in affecting health-related lifestyle changes in diabetic patients [98]. For example, diabetic patients mentioned various barriers to physical activities, including lack of willpower, energy, time, resources, or skills, social influences, and fear of injury [99].

However, for the prevention of diabetes und consequently, also DR, lipid profile, blood pressure, and glycemia can be controlled by pharmacological treatment but also by a healthy lifestyle that includes physical activity and changes dietary practices [100]. Moreover, peer support groups can improve self-management and promote healthy behavior; in fact, diabetic patients that are members of peer support groups report satisfaction and positive lifestyle change [101]. Similar community settings, including health-seeking behavioral changes or dietary arrangements, can provide positive diabetes self-management education interventions for patients [102]. The Finnish diabetes prevention study revealed that appropriate lifestyle intervention, which included weight loss (BMI reduction), a healthy diet, and physical activity, improved DR status [103]. It is also significant to note that stopping cigarette smoking can reduce the risk of DR in diabetic patients [104]. Studies on the effect of alcohol consumption on DR have reported contradictory results. One study reported that daily moderate alcohol consumption (0.5 to 1.0 mg/kg) worsened diabetic control and increased the prevalence of DR, impotence, and possibly peripheral neuropathy [105], whereas a more recent study has reported that alcohol consumption by DM patients reduced the incidence of DR compared with non-drinkers [106].

### Physical activity

A sedentary lifestyle is often associated with obesity, which often leads to diabetes [96]. A study by Bukht et al. revealed that low physical activity or physical inactivity often led to obesity and diabetes-related complications in urban Bangladeshi population [107]. We should note here that physical inactivity and sedentary behavior are modifiable risk factors for DR [108]. In fact, reduced risk of DR through physical activity was observed in a meta-analysis of twenty-two studies; moderate intensity physical activity exerted more beneficial effect on DR compared to both low- and high-intensity physical activity [109]. Similarly, higher physical activity and a lesser sedentary lifestyle were associated with a lower prevalence of DR in the population-based Beijing Eye Study [110]. Moderate-to-vigorous physical activity (aerobic-based) but not muscle-strengthening activity reduced systemic inflammation through decreased C-reactive protein levels among DR patients in the USA, suggesting that decreased inflammation can be associated with alleviation of the DR progression [111]. Another study indicated that frequent leisure-time physical activity reduced the severity of DR after adjusting for gender, duration, age at onset of diabetes, kidney function, triglycerides, systolic blood pressure, and BMI [112]. Yan et al. have shown that physical activity was related to decreased risk of retinal photocoagulation as well as DR progression in the Australian population of working-aged diabetic patients [113]. Association between physical activity and DR is influenced by various modifiers, including BMI, gender, history of CVD, diabetes (in family), and insulin therapy [113]. In addition to diabetes, retinopathy can result from retinal telangiectasia, systemic vasculitis, or macroaneurysm. A 2018 study by Frith and Loprinzi demonstrated that every 1 min/day increase in non-bouted (“lifestyle physical activities” vs. structured exercise; i.e., bouted physical activity) moderate-to-vigorous physical activity reduced the probability of moderate-to-severe retinopathy in US patients [114].

A 2017 study by Kuwata et al. reported that higher (walking, moderate, and vigorous) physical activity was related to a lower incidence of DR [115] in Japanese patients with T2D. Even though several studies have reported that various types of physical activities can reduce the risk of DR or its severity, prevalence, or progression, the detailed protective molecular mechanisms of physical activity remain unclear. Table 2 summarises data on the protective effects of physical activity on the development and progression of DR.

**Table 2:** Physical activity as preventive or mitigating tool against DR

Type of physical activity	Study details	Results	Reference
Various types of physical activity	Meta-analysis of twenty-two studies (Medline (accessed by PubMed), EmBase, and Cochrane Library)	↓ DR risk, moderate intensity of physical activities: ↑ beneficial effect on DR	[109]
Higher physical activity and less sedentary lifestyle	Population-based Beijing Eye Study: participants (*n* = 3468), individuals with information of their physical activity (*n* = 3031 (87.4%))	↓ prevalence of DR	[110]
Moderate-to-vigorous physical activity	Retinopathy patients (*n* = 157) between 40 and 85 years of age	Moderate-to-vigorous physical activity (30 min/day): ↓ 0.12 mg/dL CRP, ↓ systemic inflammation	[111]
Leisure-time physical activity	Finnish individuals with type 1 diabetes and retinopathy (*n* = 1612)	↓ severity of DR	[112]
Walking, moderate, and vigorous physical activity	All participants (*n* = 266,896), eligible participants—working-aged diabetic patients from New South Wales, Australia (*n* = 9018)	↓ risk of retinal photocoagulation, ↓ DR progression	[113]
Non-bouted physical activity	Broader US adult participants (*n* = 1501) between 40 and 85 years	↓ probability of diagnosis of moderate-to-severe retinopathy	[114]
Higher (walking, moderate, and vigorous) physical activity	Japanese patients with type 2 diabetes (*n* = 1814)	↓ incidence of DR	[115]

### Diet

High-fat or a combination of high-fat and high-sugar diet is a common risk factor of DR and is used for the induction of DR in in vivo animal models [116–118]; however, not every type of fat seems to be harmful what has to be considered when interpreting differing results in clinical studies. Alcubierre et al. demonstrated that high monounsaturated fatty acid (oleic acid) consumption was not associated with increased DR risk [119] while Sasaki et al. reported that dietary intake of polyunsaturated fatty acids was related to increased DR risk [120]. The Mediterranean diet, one of the healthiest diet in the world, is characterised by higher intake of fruits, vegetables, and fish and can protect against the development of DR [121–123].

Thus, a balanced diet and healthy lifestyle can mitigate or prevent the development of DR as well as supplementation with nutraceuticals containing vitamins (B_1_, B_2_, B_6_, B_12_, C, D, E, and l-methylfolate) and minerals (zinc) [124]. Their consumption can also support conventional therapies (Metanx^®^, Cystadane^®^, and Ocufolin^®^) that reduce DR risk or severity [124]. Nutraceuticals can reduce the neural and vascular damage in DR through their anti-inflammatory and anti-oxidant properties [125]. Per evidence, the Mediterranean diet and higher intake of fruits, vegetables, and fish in general can protect predisposed diabetics against the development of DR [121–123].

### Metformin treatment

Metformin, which is a widely used oral anti-hyperglycemic agent for the treatment of T2D, has been shown to have protective effects on retinal pigment epithelium (RPE) cells [126]. Among other effects, metformin is an AMP-activated protein kinase (AMPK) agonist, suggesting that AMPK-dependent signalling pathways are involved in the pathophysiology of DR [126].

Measurement of inflammatory cytokines in human vitreous from control individuals and patients with PDR with or without long-term metformin treatment (> 5 years) shows a decrease in PDR patients in metformin-treated compared to non-treated patients [127]. In the same study, metformin inhibited high glucose (HG)–induced ICAM-1, IL-8, and MCP-1 via AMPK activation in human retinal vascular endothelial cells (hRVECs) [127]. On the other hand, inhibition of NF-κB p65, sICAM-1, and TNF-α by metformin was not dependent on the AMPK signalling pathway. In addition, data showed that the down-regulation of the inflammatory cytokines could be affected via inhibition of NF-κB, which is independent of the AMPK pathway. Song et al*.* [128] evaluated the effect of AMPK on diabetes-induced retinopathy in animal models and showed that AMPK activation by metformin delayed diabetes-induced retinopathy by regulating mitochondrial function and reducing autophagy. Compared to non-treated control, metformin increased autophagy-related protein LC3, decreased protein p62 and Bax/Bcl-2 ratio, and increased mitochondrial-related gene expression and membrane potential [128]. Metformin has also been shown to reduce vessel formation and migration in HG-treated human microvascular endothelial cells via AMPK-independent signalling [129]. Metformin slightly increased expressions of several genes important for angiogenesis and fibrosis, i.e. *TGFß2*, *VEGFR2*, *ALK1*, *JAG1*, *TIMP2*, *SMAD5*, *SMAD6*, and *SMAD7* suggesting that metformin improves endothelial dysfunction and, therefore, might be clinically relevant preventing DR development. Indeed, in a preclinical study, metformin reduced ocular degeneration in streptozotocin-induced hyperglycemic rats. Preventive effects of metformin on vascular abnormalities in this animal model of diabetes were characterised by restoration of serum VEGF, TNF-α, glutathione/malondialdehyde, and claudin-1 in the cornea, lens, sclera, ciliary body, iris, conjunctiva, retina, and optic nerve [130]. Metformin has also been shown to suppress diabetes-induced atherosclerosis, to reduce vascular smooth muscle cell migration and infiltration in atherosclerotic plaques via the activation of the AMPK–Pdlim5 pathway [131]. The results point to metformin as a drug with considerable anti-angiogenic and anti-inflammatory activities on retinal vasculature [132]. Retinal apoptosis plays a critical role in the progression of DR: using a mouse diabetes model, Kim et al. ([133] reported that metformin decreases co-localisation of TUNEL-positive ganglion cells and O-linked β-*N*-acetylglucosamine (O-GlcNAc) transferase (OGT), carbohydrate-responsive element-binding protein (ChREBP), and thioredoxin-interacting protein, or NF-κB and suppresses interaction between OGT and ChREBP or NF-κB. The authors concluded that OGT down-regulation may be one of the mechanisms by which metformin reduces cell death in the retina. The anti-apoptotic effects of metformin in a rat model of DR were characterised by decreases in the serum levels of oxidative stress markers and caspase-3 expression [134]. AMPK-mTOR signalling can trigger autophagy in RPE cells and thus can play a crucial role in the maintenance and self-clearance of normal cellular function [135]. Metformin as an agonist of the AMPK pathway may reduce RPE cell and photoreceptor cell death in the prevention and treatment of DR [135]. In this regard, the protective effect of metformin on endothelial cells under HG conditions could be partly explained by its role in attenuating autophagy via GLI1-dependent HG signalling activation. An in-depth analysis has shown that *GLI1* knockdown–initiated autophagy was associated with an increase in BNIP3 disrupting the connection of BECN1/Beclin 1 and Bcl-2 [136]. Signalling pathways such as Hedgehog, miR-570-3p, miR-142-3p, or MiR-3127-5p could be involved in the process of metformin-inhibited autophagy [137]. Oubaha et al. [138] reported that ischemic retinal cells use the inositol-requiring enzyme 1a (IRE1a) pathway via IRE1α that initiates a senescence-associated secretory phenotype in cells (SASP). Patients suffering from PDR showed increased levels of SASP-associated cytokines (plasminogen activator inhibitor 1, IL-6, IL-8, and VEGF) in the vitreous humor [138], and intravitreal application of metformin in mice inhibited SASP and reduced the destructive retinal neovascularisation in vivo [138].

### Phytochemicals targeted to individual risks

Phytochemicals represent naturally occurring substances that are associated with biological effects, including anti-inflammatory, anti-microbial, anti-cancer, or neuroprotective, among others [139–146]. Therefore, we can assume a potential beneficial effect of plant-derived phytochemicals also in DR [147].

#### Pathomechanisms associated with DR which can be targeted by phytochemicals

Natural substances exert numerous health-beneficiary effects [139–143, 145, 146]. Therefore, here, we provide a brief overview of mechanisms related to DR stratified for individual risks factors such as degeneration, inflammation, vasculature, cell protection, and apoptosis. The early non-proliferative stage of DR is characterised by increased vascular permeability associated with the breakdown of BRB, thickening of basement membrane, and pericyte loss in retinal capillaries. In the active PDR stage, retinal neoangiogenesis results in the formation of a fragile vasculature associated with retinal injury leading to the loss of vision and blindness. Diabetic macular edema, which results in visual impairments, is also associated with vascular permeability and BRB dysfunction [148]. In addition, AGEs directly contribute to the initiation and progression of DR [149]. AGEs are defined as proteins or lipids glycated after an exposure to sugars that are widespread in diabetic vasculature. The accumulation of AGE in different cell types affects their function and structure and contributes to the micro-/macro-vascular deficiencies [150]. Also, neurodegeneration is considered an initiating factor of retinal damage leading to microvascular impairments in DR while oxidative stress induced by diabetes is considered a key factor damaging neurons of the diabetic retina [151]. Moreover, the evidence describes an association between neuronal abnormalities (neuronal cell death) and early DR. Indeed, mitochondrial- and caspase-dependent cell death and retinal neuronal cell degeneration are described in patients with diabetes [152]. In addition, inflammation per evidence is associated with DR, which is considered an inflammatory disease. Inhibition of inflammation has been shown to mitigate DR development. The activation of inflammatory cells in the microglia is observed in diabetic patients, and pro-inflammatory cytokines released by microglial cells are found in the retinae of diabetic patients [148]. Moreover, chronic inflammation that affects vascular permeability results in microvascular occlusions and ischemic signalling [153].

#### Phytochemicals targeting DR—an evidence-based contribution to the PPPM concept

The flavonoid ( −)-epicatechin, found in green tea and coca, exerts numerous health beneficial effects [154, 155]. (−)-Epicatechin has demonstrated the capacity to break preformed glycated serum albumin in vitro and to reduce AGE retinal accumulation in vivo; (−)-epicatechin treatment of exogenously AGE-injected rats resulted in improved retinal vascular apoptosis and reduced AGE burden [154]. Naringenin, a flavonoid found in citrus fruits, attenuated oxidative stress through the modulation of oxidative stress markers, especially retina glutathione (GSH) and the lipid peroxidation product thiobarbituric acid reactive substances (TBARS), and modulated apoptotic factors (Bcl-2, caspase-3, and Bax) [156]. In addition, naringenin improved neurotrophic effects in diabetic rat retina as demonstrated by increased brain-derived neurotrophic factor (BDNF) and improved levels of tropomyosin-related kinase B (TrkB) and synaptophysin [156]. Overall, the anti-diabetic, anti-oxidant, and anti-apoptotic effects of naringenin demonstrated in diabetic rats highlight its potential to ameliorate neurodegeneration through neurotropic support for preventing retinal damage in DR [156].

The flavonoid quercetin has demonstrated capacity to protect retinal neurons from damage in DR through amelioration of neurotropic factors and inhibition of apoptosis of neurons in diabetic rats [157]. Quercetin administration provided protection against DR through heme oxygenase-1 expression in a rat model of STZ-induced DR [157]. Specifically, quercetin increased the thickness of retinal cell layer and number of ganglion cells and reduced the overexpression of pro-inflammatory cytokines and high mobility group box-1 (HMGB1) [157]. Quercetin also reduced TLR4 overexpression and NF-κB p65 overactivation caused by sustained hyperglycemia, reduced NLRP3 inflammasome overactivation, and upregulated pro-angiogenic factors as well as neurotropic factors, which are essential for the protection of retinal ganglion cells [157]. However, the upregulation of NGF was not affected by HO-1 [153]. Moreover, phytochemicals exert also retinal protective effects associated with ischemia–reperfusion injury. Arikan et al. showed the capacity of quercetin to protect the retina by reducing apoptosis due to ischemia–reperfusion injury in rat retina, specifically reducing thinning of all retinal layers [158].

Administration of a flavonoid hyperoside isolated from *Hypericum* and *Crataegus* species demonstrated retinal protective effects from HG-DM through anti-oxidant effects as well as inhibition of cell damage and apoptosis in vitro and in vivo [159]. In addition, galangin, a flavonoid found in *Alpinia officinarum* belonging to the ginger family, ameliorated DR through the attenuation of BRB damage, inhibition of inflammation triggered by microglia, and suppression of BRB dysfunction induced by TNFα inhibition in vivo and in vitro [148].

NAD^+^/NADH redox balance is altered in diabetes; consequently, Fu et al. demonstrated that crude chickpea flavonoid extract relieved NAD^+^/NADH redox imbalance, alleviated mitochondrial complex I dysfunction, and reduced oxidative stress in the pancreas of a T2D rat model [160].

It has also been shown that ginger extract improves DR through the inhibition of endothelial/inducible nitric oxide (NO) synthase (e/iNOS) expression, G6PDH reduction, and decreased oxidative damage in a rat model of T2D retinopathy. G6PDH participates in the pentose phosphate pathway and produces NADPH that is associated with eNOS, NO, and maintenance of protection against oxidative damage. Ginger extract also reduced inflammation, angiogenesis, and apoptosis in the same rat model [161].

A review of the effects of polyphenols and carotenoids, by Bungau et al. showed that these phytochemicals exert beneficial effects in age-related ophthalmopathies and DR [162]. Notably, luteolin, a retinal-specific carotenoid, demonstrated retinal protective effects by preventing the upregulation of VEGF and down-regulation of superoxide dismutase 2 (SOD2) in retinae of diabetic rats and prevented abnormalities of ganglion cells in the inner and outer nuclear layer in diabetic retinae. Indeed, the authors concluded that the protective effects of luteolin are mediated via mRNA expression of HIF1α and X-box binding protein 1 (Xbp1) [163]. However, a recent study by McClinton et al. demonstrated the beneficial effects of high dose dietary carotenoids in healthy retina but detrimental effects in diabetes in vivo [164].

In addition to experimental in vitro and in vivo research, several clinical studies have evaluated the impact of phytochemicals on visual damage associated with DR. The results of a retrospective study concluded that carotenoid supplements have potential beneficial effects for improving visual function in T2D patients; however, the authors highlighted the need for further studies on long-term effectiveness [165]. In a recently published prospective case–control study, Chiosi et al. described a positive effect of fixed oral combination of curcumin, artemisinin [166] (a bioactive metabolites of Artemisia) [167], bromelain (a cysteine protease from pineapples), and black pepper on central retinal thickness, visual acuity, and vessel density in deep capillary plexus in compensated T2D patients with mild diabetic macular edema [166].

Thus, as summarised in Table 3, current scientific evidence provides results of numerous, primarily experimental studies, on the protective effects of phytochemicals on DR risk, retinal vasculature, inflammation, apoptosis, neurodegeneration, oxidative stress and mitochondrial dysfunction, and the effects of phytochemicals in age-associated ophthalmopathies. However, further investigations are needed to fully elucidate the potential effects of naturally occurring substances for the protection of visual loss in DR.

**Table 3:** Phytochemicals targeted to individual risks

Phytochemical	Study details	Affected mechanisms	Effects	Ref
Preclinical research				
( −)-Epicatechin	In vitro (bovine serum albumin)	Vasculature (AGE, vascular apoptosis)	Destroyed preformed glycated bovine serum albumin-collagen cross links	[154]
	In vitro (human serum albumin formed in diabetic patients)		↓ antigenic glycated human serum albumin	
	In vivo (exogenous AGE-modified rat serum albumin-injected rats)		↓ AGE burden and prevented apoptosis of retinal vascular cells	
Naringenin	In vivo (male Wistar albino rats; diabetes induced by STZ)	Neuroprotective factors, apoptosis, oxidative stress	↑ neuroprotective factors (BDNF, TrkB, and synaptophysin), modulation of apoptotic factors (↑ Bcl-2, ↓ Bax and caspase-3) ↓ TBARS and increased GSH vs. non-treated diabetic rats	[156]
Quercetin	In vivo (male Wistar albino rats; diabetes induced by STZ)	Neuroprotective factors, apoptosis, oxidative stress	↑ BDNF, NGF, TrkB, ↑ Akt survival pathway to almost normal relative to control, ↑ Bcl-2, ↓ caspase-3 and Bax, ↑ GSH vs. non-treated diabetic rats	[157]
Quercetin	In vivo (male Sprague–Dawley rats; diabetes induced by STZ)	Cell protection, neurodegeneration, vasculature, inflammation,	Improved histopathological changes (↑ thickness of INL, ONL and ↑ distance between ILM and OLM); ↑ ganglion cells; ↓ pro-inflammatory cytokines (IL-1β, IL-18, IL-6, TNF-α), synchronous ↓ of HO-1 blocked reduction of pro-inflammatory cytokines by quercetin; ↓ HMGB1 and NLRP3 inflammasome-related proteins (by inducing HO-1); ↓ TLR4 overexpression and NF-κB p65 overactivation in HO-1 dependent manner; ↑ pro-angiogenic VEGF and sICAM-1; ↑ neurotropic factors BDNF in retina in HO-1-dependent manner and ↑ NGF	[153]
Quercetin	In vivo (ischemia–reperfusion rat model)	Apoptosis	↓ thinning of all retinal layers (INL, ONL); ↓ apoptosis (↓ number of TUNEL ( +) cells and caspase-3 ( +) cells significant in INL)	[158]
Hyperoside	In vitro (RVECs); in vivo (male Sprague–Dawley rats; diabetes induced by STZ)	Cell protection, apoptosis, oxidative stress	↓ RVECs damage, oxidative stress, and apoptosis (in vitro); ↓ blood glucose, pathological damage of retina; ↑ proliferation of RVECs, ↓ apoptosis; ↓ caspase-3, ↓ caspase-9, ↓ and Bax in RVECs; ↑ Bcl-2 (in vivo)	[159]
Galangin	In vivo (C57BL/6 male mice; diabetes induced by STZ), in vitro (HREC, microglia BV2 cells)	Inflammation, BRB damage	↓ BRB damage and retinal inflammatory injury, ↓ microglia cell activation (in vivo); ↓ d-glucose-induced BV2 cell activation, ↓ activation of NFκB and Egr1, suppression of RK1/2 phosphorylation and ROS in D-glucose induced BV2, ↓ D-glucose-stimulated BV2 cell-mediated BRB-damage, ↓ TNFα-induced BRB damage	[148]
CCFE	In vivo (male Sprague–Dawley rats, type 2 diabetes mellitus model)	Redox balance, mitochondrial dysfunction, oxidative stress	↓ NAD^+^ /NADH redox imbalance, ↓ mitochondrial complex I dysfunction (complex I activity suppression), ↓ oxidative stress (↓H_2_O_2_, MDA and ↑GSH-Px, SOD)	[160]
Ginger extract	In vivo (male Wistar rats, T2D induced by high-fat diet)	Apoptosis, inflammation, angiogenesis, oxidative stress	Improved TNF-α and oxidative stress biomarkers; improvement of genes in ocular tissue of diabetic rats (↓ VEGF, NF-κB, iNOS, Bax, and caspase-3; ↑ Bcl-2, eNOS, and G6PDH)	[161]
Luteolin	In vivo (male Wistar rats; diabetes induced by STZ)	Angiogenesis; oxidative stress	↓ VEGF, HIF1α, Xbp1; ↑ SOD2; ↓ GSH; prevention of ganglion cell loss; maintenance of INL intact	[163]
Carotenoid-rich carrot powder	In vivo (male Wistar rats; diabetes induced by STZ)	Retina function	Non-diabetic rats: ↑ rod- and cone-driven post-synaptic b-wave amplitudes vs. rats with control diet; diabetic rats: exacerbated retina dysfunction (↓ amplitudes of most rod- and cone-driven electroretinogram components)	[164]
Clinical research				
Carotenoid supplementation: lutein (10 mg), zeaxanthin (2 mg), meso-zeaxanthin (10 mg) once/day for 2 years	Retrospective study; patients with non-insulin type 2 diabetes mellitus without retinopathy (*n* = 60)	Visual function	↑ central foveal thickness and retinal response density)	[165]
Fixed combination of curcumin (200 mg), artemisinin (80 mg,) bromelain (80 mg), and black pepper (2 mg)	Prospective case–control study; patients with type 2 diabetes mellitus (*n* = 56)	Positive effects on mild DME in compensated T2D patients (non-proliferative DR)	Improved BCVA and CRT reduction vs. control	[166]

*Abbreviations: Bax* Bcl-2-associated X protein, *Bcl-2* B cell lymphoma 2, *BCVA* best corrected visual acuity, *BDNF* brain-derived neurotropic factor, *CCFE* crude chickpea flavonoid extract, *CRT* central retinal thickness, *DME* diabetic macular edema, *DR* diabetic retinopathy, *eNOS* endothelial NO synthase, *G6PDH* glucose-6-phosphate dehydrogenase, *GSH* glutathione, *GSH-Px* glutathione peroxidase, *H*_*2*_*O*_*2*_ hydrogen peroxide, *HMGB1* high mobility group box-1, *HO-1* heme oxygenase-1, *HRECs*, primary human retinal endothelial cells, *ILM* inner limiting membrane, *INL* inner nuclear layer, *iNOS* inducible NO synthase, *MDA* malondialdehyde, *NF-κB* nuclear factor-kappaB, *NGF* nerve growth factor, *NLRP3* NLR family pyrin domain containing 3, *OLM* outer limiting membrane, *ONL* outer nuclear layer, *RVECs* retinal vascular endothelial cells, *sICAM-1* soluble intercellular cell adhesion molecule-1, *SOD* superoxide dismutase, *SOD2* superoxide dismutase 2, *STZ* streptozotocin, *TBARs* thiobarbituric acid reactive substances, *TLR4* Toll-like receptor 4, *TrkB* tropomyosin related kinase B, *VEGF* vascular endothelial growth factor, *Xbp1* X-box binding protein 1

## Conclusions and recommendations in the framework of predictive, preventive, and personalised (3P) medicine

### Population screening for people in a state of suboptimal health is strongly recommended

The current screening process for DR risk is not effective enough, and thus, often, the disease develops and progresses undetected until irreversible damage occurs. Small vessel disease and neuroretinal changes create a vicious circle in the development of PDR with characteristic systemic attributes including excessive mitochondrial and retinal cell death, chronic inflammation, neovascularisation, and impaired visual field leading to blindness. Moreover, PDR is considered an independent predictor of other severe diabetic complications such as ischemic stroke. Thus, effective population screening at the level of suboptimal health conditions, defined as an intermediate health status between health and illness [168], is strongly recommended to identify and quantify reversible damage to health for a targeted protection against DR and further even life-threatening complications of diabetes.

### Mitochondrial health quality control is clinically relevant in DR management

Mitochondrial injury and excessive autophagy are characteristic for the DR pathophysiology. The mechanism of progressing damage is a “vicious circle” by ROS overproduction accompanied with a diminished energy production and extensive oxidative stress to mtDNA and chrDNA [69]. The severity is measurable via the mitochondrial health quality control [68]. This analytical tool is of great clinical utility to promote a paradigm shift from reactive to 3P medicine in overall DR management by quantifying individual disease predisposition. Corresponding evidence-based mitigation measures are discussed in the article and elsewhere [169] such as targeting oxidative damage to mitochondria.

### Tear fluid analysis as innovative non-invasive tool for DR prediction and prognosis

Tear fluid analysis has been demonstrated as a useful tool for the diagnosis and therapy monitoring of systemic diseases with ocular complications as DR [80, 170]. Endothelial activation, inflammation, and oxidative stress attributable for DR are reflected in specific biomarker patterns detectable in the tear fluid [80]. Moreover, analysing specific molecular pattern in the tear fluid, it will be feasible.

- To stratify diabetic patients towards potential complications
- To distinguish between NPDR and PDR developing in patients with diabetic history
- To predict progressing retinal damage indicative for severe complications [9, 80].

This high analytical capacity and the fact that it can be collected non-invasively and with almost no specific training make the tear fluid a highly attractive tool in promoting the paradigm change to 3PM in DR prevention and management.

## Data Availability

Not applicable.

## Abbreviations

AGE: Advanced glycation end products
AMPK: AMP-activated protein kinase
BRB: Blood-retinal barrier
CAD: Coronary artery disease
ChREBP: Carbohydrate-responsive element-binding protein
CSVD: Cerebral small vessel disease
CVD: Cardiovascular disease
DFS: Diabetic foot syndrome
DFU: Diabetic foot ulcers
DM: Diabetes mellitus
DPN: Diabetic peripheral neuropathy
DN: Diabetic nephropathy
DR: Diabetic retinopathy
ET-1: Endothelin-1
HF: Heart failure
HG: High glucose
IDF: International Diabetes Federation
NCDs: Noncommunicable diseases
NDR: Non-diabetic retinopathy
NO: Nitric oxide
NPDR: Non-proliferative diabetic retinopathy
PDR: Proliferative diabetic retinopathy
ROS: Reactive oxygen species
RPE: Retinal pigment epithelium
T1D: Type 1 diabetes
T2D: Type 2 diabetes
WHO: World Health Organization
